# Synthesis and preclinical evaluation of a ^89^Zr-labelled human single chain antibody for non-invasive detection of hepatic myofibroblasts in acute liver injury

**DOI:** 10.1038/s41598-023-50779-w

**Published:** 2024-01-05

**Authors:** Toni A. Pringle, Erik Ramon-Gil, Jack Leslie, Fiona Oakley, Matthew C. Wright, James C. Knight, Saimir Luli

**Affiliations:** 1 School of Natural and Environmental Sciences, Newcastle University, Bedson Building, Newcastle upon Tyne, NE1 7RU UK; 2 Newcastle Fibrosis Research Group, Biosciences Institute, Newcastle University, Newcastle upon Tyne, UK; 3 Liver Research Group, Translational and Clinical Research Institute, Newcastle University, Newcastle upon Tyne, UK; 4 Newcastle Centre for Cancer, Newcastle University, Newcastle upon Tyne, UK; 5 Preclinical In Vivo Imaging, Translational and Clinical Research Institute, Newcastle University, Newcastle upon Tyne, UK; 6 Medical School, Newcastle University, 4th Floor William Leech Building, Newcastle upon Tyne, NE2 4HH UK

**Keywords:** Biomarkers, Biomarkers, Translational research, Preclinical research, Liver diseases, Hepatology, Liver, Hepatic stellate cells, Molecular imaging, Optical imaging, Positron-emission tomography

## Abstract

Synaptophysin is expressed on fibrogenic hepatic myofibroblasts. C1–3 is a single chain human antibody (scAb) that binds specifically to synaptophysin on hepatic myofibroblasts, providing a targeting vector for novel in vivo imaging agents of chronic liver disease. C1–3 and a negative control scAb, CSBD9, were radiolabelled with zirconium-89 via desferrioxamine chelation to enable non-invasive molecular imaging with positron emission tomography (PET). DFO-scAb conjugates were characterised by gel electrophoresis (SDS-PAGE) and MALDI-TOF spectrometry, and ^89^Zr-labelled with high radiolabelling efficiency (99%). [^89^Zr]Zr-DFO-C1–3 exhibited high in vitro stability (> 99%) in mouse and human sera over 3 days at 25 and 37 °C. Activated hepatic myofibroblasts incubated with [^89^Zr]Zr-DFO-C1–3 displayed significantly higher internalised activity (59.46%, P = 0.001) compared to the [^89^Zr]Zr-DFO-CSBD9 control, indicating synaptophysin-mediated uptake and high binding specificity of [^89^Zr]Zr-DFO-C1–3. Mice with CCl_4_-induced acute liver damage exhibited significantly higher liver uptake of [^89^Zr]Zr-DFO-C1–3, compared to controls, confirmed by both Cerenkov imaging and ex vivo gamma counting (4.41 ± 0.19%ID/g, P < 0.0001). CCl_4_-induced liver damage and the number of hepatic myofibroblasts was confirmed by αSMA staining of liver sections. These findings indicate that [^89^Zr]Zr-DFO-C1–3 has promising utility as a PET imaging agent for non-invasive detection of hepatic myofibroblasts following acute liver injury.

## Introduction

Chronic liver disease (CLD) is a major global health burden, accounting for approximately 2 million deaths per year^1^. The aetiology of CLD is broad and although chronic viral infections such as hepatitis B and C are a major cause of CLD^2^, the epidemiology is changing to reflect an increased prevalence of poor diet and excessive alcohol intake. Metabolic dysfunction-associated steatotic liver disease (MASLD) formerly known as non-alcoholic fatty liver disease (NAFLD) is now the leading cause of CLD, with prevalence forecasted to increase over the coming years^3–5^. It is estimated that by 2030 the incidence of decompensated liver cirrhosis and hepatocellular carcinoma (HCC), in the United States will increase by 168% and 137%, respectively^3^.

CLD is associated with hepatocyte injury which initiates an inflammatory response, leading to fibrogenesis^6,7^. In response to injury, hepatic stellate cells (HSC), typically quiescent in the normal liver, transdifferentiate into scar producing hepatic myofibroblasts (HM)^8^. To repair tissue damage, HM produce collagenous extracellular matrix forming scars which resolve when the injury stops, with HM clearing by inactivation or apoptosis^9^. However, in CLD, HM are persistently active and proliferative, driving collagen accumulation, scar formation, fibrosis, cirrhosis and eventually HCC^10^.

Early diagnosis of liver fibrosis is challenging as most patients are asymptomatic. Undetected fibrosis can progress to end-stage liver disease, including cirrhosis and increases the risk of developing liver cancer, most commonly HCC^11^. Therefore, there is a need for early diagnosis of liver fibrosis to prevent irreversible progression of liver disease. Liver biopsy is the primary method for diagnosing and staging liver disease, however, liver biopsy is an invasive and painful procedure which can cause bleeding, infection, and, in rare cases (0.01%), death^12–14^. Liver biopsy has increasingly been criticised for its lack of reproducibility caused by several factors^15^. Only 1/50,000th of the liver is sampled and so the biopsy may not be representative of disease^16^. Reproducibility is further hampered by disparities in the subjective inter- and intra-observer interpretation of results by pathologists^17^. Patient-friendly alternatives to biopsy, such as serum biomarkers, have been explored in the clinic. However, non-liver specific underlying conditions may influence the level of biomarkers in serum, providing false positive or negative results^18,19^. Shear wave elastography (SWE) and magnetic resonance elastography (MRE) have also been used in the clinic to indirectly assess liver fibrosis by measuring the mechanical properties of the liver such as tissue stiffness which arises from excessive collagen deposition and scarring^20–24^. The sensitivity of both SWE and MRE is therefore dependent on the level of scar formation, making it challenging for these modalities to detect early stage fibrogenesis and differentiate disease stages. Medical conditions such as inflammation and ascites are known increase liver stiffness, providing false-positive imaging data indicative of liver fibrosis^25^. In addition, liver stiffness has been found to significantly increase in healthy volunteers following food intake^26^.

In preclinical settings, progression of liver fibrosis is assessed histologically by humanely euthanising animals at different stages of the disease development. To overcome the challenges associated with liver biopsy and to reduce animal use in line with the 3Rs, several imaging techniques have been developed. In murine models of liver injury, studies have focused on directly assessing fibrosis by using collagen-targeted peptides labelled with MRI contrast agents^27–29^. In contrast, our group has focused on the role of HM as the primary effector cells in driving liver fibrosis^30^. Compared to other resident liver cell types, HM are characterised by the upregulation of the transmembrane protein synaptophysin (SYN)^31^. Previous reports confirm that the single chain antibody (scAb) fragment, C1–3, specifically targets SYN expressed on HM^32^. Fluorescently labelled C1–3 has previously been used to assess chronic liver injury using in vivo optical imaging, however, this technique is not clinically translatable and is currently limited to preclinical research^33^.

This study aimed to determine if C1–3 labelled with the positron emission tomography (PET) radioisotope, zirconium-89 (^89^Zr), could be used as a clinically relevant non-invasive liver fibrosis imaging agent. Here, we report the synthesis and evaluation of a zirconium-89 labelled C1–3 radioimmunoconjugate, and demonstrate that [^89^Zr]Zr-DFO-C1–3 successfully differentiates between acute liver injury and controls based on the number of HM.

## Materials and methods

### General methods

All reagents were purchased from Thermo Fisher Scientific unless otherwise stated and used without further purification. Water was deionized using a Select Fusion ultrapure water deionisation system (Suez) and had a resistance of > 18.2 MΩ cm^–1^ at 25 °C. Protein concentration measurements were obtained using a NanoDrop One Microvolume UV–Vis Spectrophotometer (NanoDrop Technologies, Inc.). MALDI-TOF mass spectrometry measurements were taken on a Bruker Microflex LRF. Radioactivity measurements were obtained using a CRC-25 Dose Calibrator (Capintec, Inc.) or a Wizard 2480 Gamma Counter (PerkinElmer). Radioimmunoconjugate synthesis and serum stability studies were monitored by instant thin-layer chromatography using glass microfiber chromatography paper (iTLC-SA, Agilent). Radio-iTLC strips were measured by autoradiography (Amersham Typhoon Bioimager, GE) and analysed using ImageQuant software (GE Healthcare).

### C1–3 production and purification

C1–3 and CSBD9 were synthesised and purified as previously described^32,34^. Briefly, *Escherichia coli* (*E. coli*) XL-1 blue cells were used to express C1–3 and CSBD9 single chain antibodies (scAb). *E. coli* were cultured over a few days in Luria–Bertani broth and Terrific Broth media containing selective antibiotics (50 μg/mL of ampicillin and tetracycline). Expression of the antibodies was induced by adding 0.5 M Isopropyl β-d-1-thiogalactopyranoside (IPTG) followed by a 4-h culture. To isolate C1–3 and CSBD9, cells were resuspended in a fractionation buffer (200 mM Tris–HCl, 20% sucrose, 1 mM EDTA, pH 7.5) supplemented with 50 mg/L lysozyme to lyse the cells. Following successful cells lysis, C1–3 and CSBD9 were purified using immobilised metal ion chromatography (IMAC) as previously described^32,35^. Endotoxin removal was achieved using Vivapure® Maxi H spin Q columns (Sartorious Vivascience).

### Synthesis of ScAb-DFO

C1–3 and CSBD9 scAb fragments (400 µg, 1 mg/mL, PBS) were washed and concentrated in pre-rinsed 10 kDa molecular weight cut-off 0.5 mL centrifugal filters (Amicon) at 14,000×*g* for 15 min and washed three times (3 × 500 μL, 0.1 M NaHCO_3_, pH 8.9). To the purified scAb solutions was added a 2.5-fold molar excess of p-SCN-Bn-DFO (2 mg/mL in DMSO, Macrocyclics) before briefly vortexing, centrifuging, and incubating at 37 °C and 450 rpm for 1 h. The DFO-C1–3 and DFO-CSBD9 solutions were then purified by centrifugal filtration as previously described and diluted to 2 mg/mL with PBS (pH 7.2). scAb concentrations were determined by UV–Vis spectroscopy (Nanodrop One Spectrophotometer) with ε_molar_ = 54,610 and MWT = 37,777.99 Da.

### MALDI-TOF

A saturated solution of α-cyano-4-hydroxycinnamic acid (αCHCA, 20 mg) in acetone (500 µL) was prepared as Mix 1. A precursor saturated solution of CHCA (20 mg) in 70% acetonitrile with 5% formic acid (500 µL) was prepared alongside a saturated solution of 2,5-dihydroxybenzoic acid (DHB, 20 mg, Sigma-Aldrich) in 70% acetonitrile with 0.1% trifluoroacetic acid (500 µL). Solutions were prepared at room temperature and vortexed thoroughly for 60 s before use. DHB (100 µL) and CHCA (100 µL) solutions were then combined to prepare Mix 2. Mix 1 (0.5 µL) was spotted onto a polished steel target plate (Bruker) and evaporated quickly to leave a thin layer of CHCA. A 0.5 µL aliquot of protein sample (typically 10 µM in PBS) was spotted directly onto the layer. Then, 0.5 µL of Mix 2 was added to the liquid droplet and allowed to dry.

A Bruker Microflex LRF was used to acquire the MALDI–TOF–MS data, in linear positive mode (laser 60 Hz, Ion Source 1: 19.5 kV, Ion Source 2: 18.15 kV, Lens: 7.00 kV, Pulsed Ion Extraction 240 ns, Detector Gain 2850 V). Data were processed using Bruker flexAnalysis v3.4.

### Radiolabelling

Zirconium-89 in oxalic acid (PerkinElmer) was adjusted to pH 7 by the addition of 1 M Na_2_CO_3_ (assessed by pH indicator paper). Neutralised ^89^Zr was added to 2 mg/mL solutions of DFO-C1–3 and DFO-CSBD9 (100–150 µg) to achieve a specific activity of 0.05 MBq/µg. The reaction mixture was incubated for 1 h at 25 °C and 450 rpm. The radiolabelling efficiency was assessed by radio-iTLC using 50 mM EDTA (pH 5.5) as the mobile phase. The radioimmunoconjugates were used without further purification where radiochemical purity was > 99%.

### Radio-SDS-PAGE

C1–3 conjugates were analysed by sodium dodecyl sulfate–polyacrylamide gel electrophoresis (SDS-PAGE). Protein samples (1 µL, 1 mg/mL) were prepared by the addition of NuPAGE 4× LDS sample buffer (2.5 µL) and deionised water (6.5 µL) to a total volume of 10 µL. The resulting solutions were incubated at 70 °C for 10 min at 400 rpm. Protein samples and molecular weight standards (ThermoScientific PageRuler Unstained Broad Range Protein Ladder, 10 µL) were loaded on a 10-well protein gel (4–12% Bis–Tris) and resolved for 40 min at 200 V in NuPAGE MOPS SDS running buffer. The gel was subsequently washed three times in deionised water (100 mL, 5 min) then stained with Coomassie Fluor Orange (50 mL, Invitrogen) for 1 h with gentle rocking. Destaining was performed by soaking the gel in 1 M acetic acid (50 mL) for 1 min, then washing with deionised water (250 mL, 5 min). The gel was scanned using a Typhoon Bioimager for Coomassie Fluor Orange (λ_ex/em_ = 488/525 nm) and autoradiography.

### Serum stability

[^89^Zr]Zr-DFO-C1–3 (1.80 µg, 1 µL, 0.05 MBq/µg) was added to human serum (50 µL, Sigma-Aldrich, Cat# H4522), mouse serum (50 µL, Sigma-Aldrich, Cat# M5905) and PBS (50 µL, pH 7.2) in triplicate and incubated at 25 and 37 °C. Stability was assessed every 24 h over 3 days by radio-iTLC using the aforementioned conditions.

### Hepatic stellate cell (HSC) isolation and culture activation

Primary mouse hepatic stellate cells (HSCs) used in this manuscript were freshly isolated from the livers of wild type C57BL/6 mice by collagenase and pronase digestion. Following enzymatic digestion, HSC were separated from other liver cell types using an Optiprep (Sigma-Aldrich, Cat# 92339-11-2) density gradient and elutriation as previously described^36–38^. HSCs were seeded into a 75 mL plastic flask and cultured in Gibco Dulbecco’s Modified Eagle Medium (DMEM, Gibco, Cat# 41965039) supplemented with 16% foetal bovine serum (FBS, Sigma-Aldrich, Cat# F9665) and penicillin/streptomycin (10 mg/L). HSCs were culture-activated over a period of 10 days to achieve maximum transdifferentiation into HM.

### Cellular internalisation assay

HM were subcultured when they reached 85–95% confluency through removal of old media, washing with PBS (pH 7.2), and lifting cells using trypsin/EDTA (Sigma-Aldrich, Cat# T4049).

Cells were seeded in 0.5 mL media in 24-well plates at an appropriate density to achieve 40,000 cells/well at the end of the assay, 1 day prior to performing the assay. [^89^Zr]Zr-DFO-C1–3 and [^89^Zr]Zr-DFO-CSBD9 diluted in media (300 µL, 60 µg/mL) were added to wells in triplicate. Two hours after incubation, the supernatant media (free fraction) was transferred to allocated counting tubes. Cells were washed with 0.5 mL of PBS (pH 7.2) which was then combined with the respective supernatant media. Cells were then incubated with cold 0.1 M glycine HCl (0.5 mL, pH 2.5, Sigma-Aldrich) for 10 min which was then transferred to a separate series of counting tubes (membrane-bound fraction) and combined with 0.5 mL of PBS (pH 7.2) used to wash the cells. Lastly, RIPA lysis and extraction buffer (0.1 mL, ThermoScientific) was added to each well and plates were placed on ice for 5 min before transferring the cell lysates (internalised fraction) to a third series of counting tubes, followed by the addition of a 0.5 mL PBS (pH 7.2) cell wash. The activity (counts per min, CPM) in each counting tube was measured on a gamma counter, converted to activity units (MBq) using recent calibration data, and decay corrected to the start of the experiment.

### Mouse model

All mouse experiments were carried out in accordance with UK Animals (Scientific Procedures) Act, 1986 under UK home office license following approval by the Newcastle University Animal Welfare Ethical Review Body (AWERB) and in accordance with the ARRIVE guidelines.

#### Acute carbon tetrachloride liver injury

Carbon tetrachloride (CCl_4_) is a hepatoxic agent commonly used to induce acute and chronic liver injury. Liver injury in C57BL/6 male mice was induced with a single dose of CCl_4_. CCl_4_ was prepared in olive oil vehicle (CCl_4_: olive oil, 1:1 [v/v]) and administered intraperitoneally (IP) at 2 µL/g of body weight. Control mice received a single IP injection of olive oil at 1 µL/g of body weight.

### In vivo Cerenkov luminescence imaging

To induce activation of HM, leading to the expression of synaptophysin, mice were injected IP with CCl_4_. Control mice were injected with oil vehicle. At 48 h post injury, mice were injected intravenously with either [^89^Zr]Zr-DFO-C1–3 or control [^89^Zr]Zr-DFO-CSBD9 (0.89 ± 0.04 MBq, 9.83 ± 0.45 µg, 100 µL).

At 72 h post-injury, mice underwent in vivo Cerenkov luminescence imaging (CLI) using an optical in vivo imaging system (IVIS Spectrum, Caliper Life Sciences). Images were acquired using the following parameters: Excitation Filter = block, Emission Filter = Open, Binning = 8, Field of view = 13.1 × 13.1 cm, f-Stop = 1, Exposure = Auto (max 300 s).

### Ex vivo gamma counting

Following in vivo imaging, mice were humanely killed and organs were harvested. Organs were transferred into pre-weighed counting tubes, weighed and the activity in each sample was measured with a gamma counter. Counts per minute were converted into activity units (MBq) using a calibration curve generated from known standards. These values were decay corrected to the time of injection, and the percentage of the injected dose per gram (%ID/g) of each liver was calculated. Livers were then flash-frozen with dry ice and stored at − 80 °C until required for further processing.

### Alpha smooth muscle actin (αSMA) histological staining

Mouse liver tissue was fixed for 24 h in 10% formalin in PBS. Paraffin-embedded liver sections were cut at 5 µm thickness. Following dewaxing, endogenous peroxidase activity was blocked with 2% hydrogen peroxide in methanol. Antigen retrieval was performed using citrate based unmasking solution (Vector Laboratories). Following avidin/biotin and serum blocking, liver sections were incubated overnight at 4 °C with anti-α-SMA primary antibody (1:1000, Sigma-Aldrich, Cat# F3777). After 24 h, the slides were incubated with biotinylated goat anti-fluorescein secondary antibody (1:300, Vector Laboratories, Cat# BA-0601) and followed by Vectastain Elite ABC Reagent. α-SMA antigen was visualised using a DAB peroxidase substrate kit and counterstained with Mayer’s haematoxylin. Sections were analysed at × 10 magnification using a Nikon Eclipse Upright microscope and NIS-Elements BR analysis software.

### Statistical analysis

All statistical analyses were performed using GraphPad Prism v9 (GraphPad Software, San Diego, CA, USA). A confidence interval of 95% (P < 0.05) was considered statistically significant. Student t-tests were used to compare two groups. One-way ANOVA followed by Tukey’s post hoc test was used to compare multiple groups. All data were obtained in at least triplicate, and results reported and graphed as mean ± standard error of the mean (SEM), unless stated otherwise. Statistical significance is shown as an asterisk where ns = not significant, *P < 0.05, **P < 0.01, ***P < 0.001, ****P < 0.0001.

## Results

### Synthesis and characterisation of scAb conjugates

**Figure 1:**
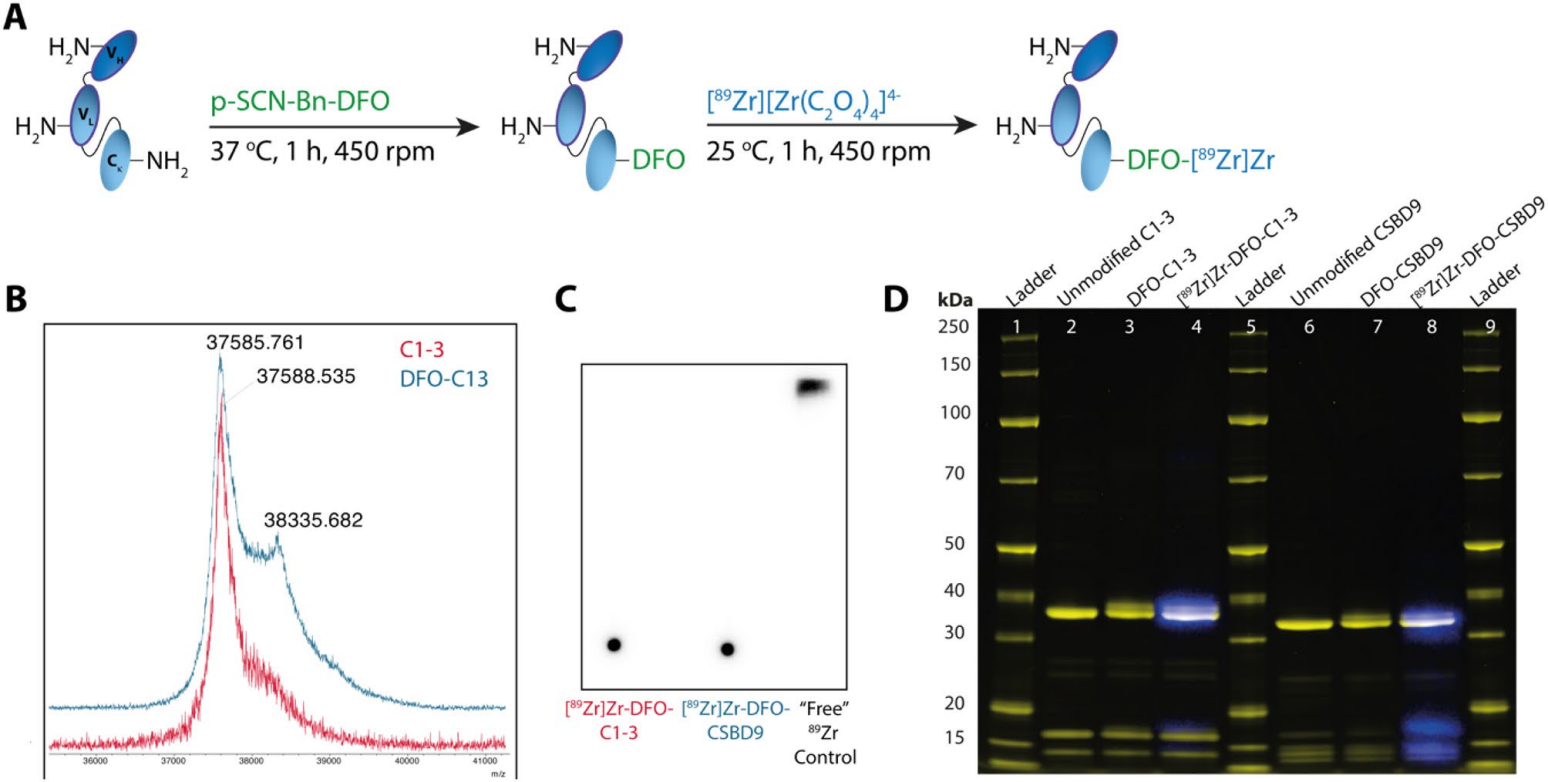
(**A**) Synthesis of [^89^Zr]Zr-DFO-scAb via conjugation of p-SCN-Bn-DFO to lysine residues (depicted as –NH_2_) followed by radiolabelling with ^89^Zr. (**B**) MALDI-TOF analysis showing an additional peak in the spectra of DFO-C1–3 (blue) compared to C1–3 (red), indicative of DFO conjugation. (**C**) Radio-iTLC of [^89^Zr]Zr-DFO-C1–3 (left), [^89^Zr]Zr-DFO-CSBD9 (middle) and a “free” ^89^Zr control (right). (**D**) Radio-SDS-PAGE of C1–3 and CSBD9 conjugates showing Coomassie Fluor Orange protein stain (yellow) and autoradiography (blue) as a co-registered image.

To facilitate radiolabelling of C1–3 and CSBD9 with ^89^Zr, each scAb was modified using an isothiocyanate derivative of the chelating agent desferrioxamine (p-SCN-Bn-DFO) via amine-directed thiourea formation in yields of 77.9 ± 13.7% (n = 2) and 76.2 ± 0.5% (n = 2) respectively (Fig. 1A). The conjugation of DFO to C1–3 was assessed by MALDI-TOF analysis, which revealed two distinct peaks in the spectrum of C1–3-DFO (37,585.76 and 38,335.68 Da). The first peak corresponds to unmodified C1–3 (37,588.54 Da) and the second peak in the spectrum corresponds to the addition of a DFO moiety, as evidenced by an increase in mass of 749.92 Da compared to unmodified C1–3 (calculated expected mass increase: ~ 755 Da, Fig. 1B). A small shoulder in this spectrum may indicate species present with multiple DFO groups attached, however the broad nature of this shoulder and the absence of a discernible peak prevent quantitative interpretation. Following modification of C1–3 and CSBD9 with DFO, the conjugates were ^89^Zr-labelled with radiolabelling efficiencies of 99.5 ± 0.7% (n = 2) and 99.5 ± 0.7% (n = 2) respectively and were used without further purification (Fig. 1C). Radio-SDS-PAGE analysis performed after completion of radiolabelling showed the expected colocalisation of radioactivity with the band corresponding to the intact scAb, and the absence of bands at higher molecular weights indicated no significant aggregate formation (Fig. 1D, Supplementary Fig. S1). While SDS-PAGE is a widely used method for monitoring the formation of protein aggregates, it is important to note this finding was not corroborated with HPLC analysis.

**Figure 2:**
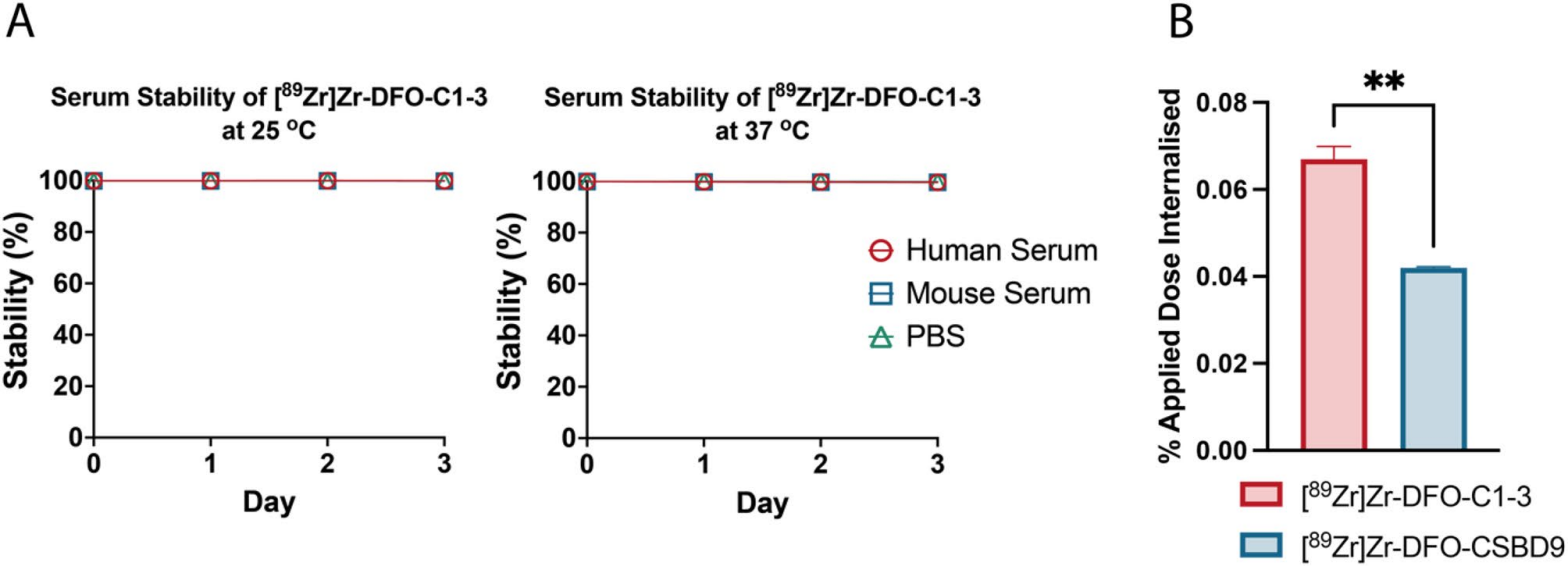
(**A**) Solution stability analysis of [^89^Zr]Zr-DFO-C1–3 over 3 days at 25 and 37 °C in human serum (red), mouse serum (blue) and PBS (green). (**B**) Uptake (percentage of applied dose) in activated hepatic myofibroblasts following a 2 h incubation of [^89^Zr]Zr-DFO-C1–3 (red) or [^89^Zr]Zr-DFO-CSBD9 (blue). Data are mean ± SEM, minimum of n = 3.

Following ^89^Zr radiolabelling, the solution stability of [^89^Zr]Zr-DFO-C1–3 and [^89^Zr]Zr-DFO-CSBD9 was assessed at both 25 and 37 °C. Radio-iTLC analysis revealed that both scAbs remained > 99% intact in human and mouse serum and PBS over a 3-day period, with no evidence of radiometal dissociation (Fig. 2A).

### In vitro evaluation: [^89^Zr]Zr-DFO-C1–3 is specifically uptaken by HM

To assess the impact of chemical modification upon binding selectivity, a cell uptake assay was performed on activated HM, characterised by upregulated SYN expression. Gamma counting analysis showed significantly higher internalisation of [^89^Zr]Zr-DFO-C1–3 in HM cells compared to the [^89^Zr]Zr-DFO-CSBD9 control (P = 0.0011, Fig. 2B), indicating that SYN binding specificity was maintained following the radiolabelling procedure.

### In vivo evaluation: [^89^Zr]Zr-DFO-C1–3 allows non-invasive imaging of HM

**Figure 3:**
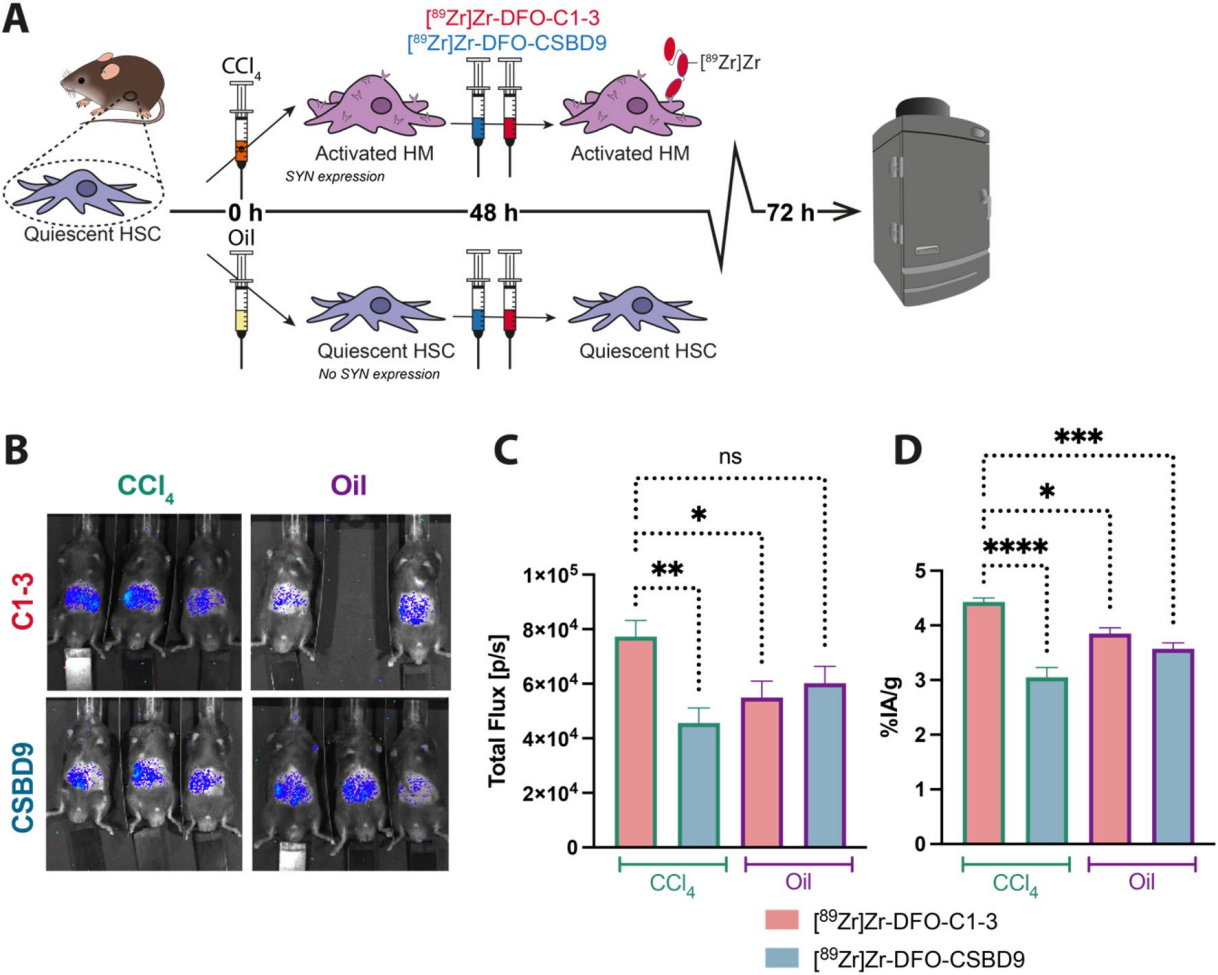
In vivo Cerenkov luminescence imaging (CLI) of [^89^Zr]Zr-DFO-C1–3 non-invasively monitors hepatic myofibroblasts in a mouse model of acute liver injury. (**A**) Schematic diagram illustrating the experimental design. Wild type C57BL/6 mice were injured with an acute dose of CCl_4_ (2 µL/g) to induce activation of HM and SYN expression. The control group was I.P administered with olive oil which does not provoke HM activation or expression of SYN. 48 h post injury, mice were administered with 0.89 ± 0.04 MBq of either [^89^Zr]Zr-DFO-C1–3 or [^89^Zr]Zr-DFO-CSBD9 via IV injection followed by in vivo CLI. (**B**) Representative IVIS in vivo CL images showed that unlike the CSBD9 control, [^89^Zr]Zr-DFO-C1–3 discriminated between injured and olive oil-treated mice. (**C**) Graph showing quantified CL images (Total Flux [p/s]), generated using regions of interest method of analysis. (**D**) Ex vivo liver gamma counting revealed that uptake of [^89^Zr]Zr-DFO-C1–3 was significantly higher compared to control groups. Data are mean ± SEM, minimum of n = 3.

After confirming the binding specificity of [^89^Zr]Zr-DFO-C1–3 for SYN was retained in vitro, the ability of [^89^Zr]Zr-DFO-C1–3 to non-invasively monitor HM activity was assessed in a mouse model of acute liver injury. To this end, liver damage was induced in wild type C57BL/6 mice via an intraperitoneal injection of CCl_4_ to induce hepatocyte death. In response to injury, HM activate and are characterised by significant upregulation of SYN^31^. The activity profile of HM shows cell numbers reaching a maximum between 48 and 72 h after injury, which are subsequently cleared 5 days after the termination of injury^30^. To maximise the binding efficiency of C1–3 to SYN, mice were intravenously injected with [^89^Zr]Zr-DFO-C1–3 or [^89^Zr]Zr-DFO-CSBD9 at 48 h post injury and imaged using an in vivo imaging system (IVIS) to detect Cerenkov luminescence at 6 and 24 h after scAb administration (Fig. 3A). Here, Cerenkov luminescence imaging was used as an alternative to PET imaging due to the lack of a preclinical PET scanner in our institute. Nevertheless, CLI provides a simple and cost-effective method to evaluate the biodistribution of radiotracers in vivo, although tissue attenuation of CL limits absolute quantification^39^.

To elucidate the clearance profile of [^89^Zr]Zr-DFO-C1–3, mice underwent CLI at 6 and 24 h post-scAb administration. At 24 h, we observed a ~ 30–60% reduction in whole body total flux compared to equivalent measurements at 6 h for all groups. The highest flux at 24 h was observed in CCl_4_-treated mice that received [^89^Zr]Zr-DFO-C1–3, indicating that SYN-specific binding of [^89^Zr]Zr-DFO-C1–3 reduces clearance rate (Supplementary Fig. S2A).

At 24 h, in vivo examination of liver uptake revealed that [^89^Zr]Zr-DFO-C1–3 can differentiate between the livers of mice with CCl_4_-induced injury, marked by an increased count of active hepatic myofibroblasts (HM) and elevated expression of SYN, and the livers of control mice treated with olive oil. The oil controls do not exhibit hepatocyte damage, HM activation, or an upregulation of hepatic SYN. To assess whether the hepatic uptake of C1–3 is SYN-specific or due to impaired clearance of the injured liver, [^89^Zr]Zr-DFO-CSBD9 was used as a control. Whole mouse body CLI showed significantly higher uptake of [^89^Zr]Zr-DFO-C1–3 in the upper quadrant of the abdominal area, corresponding to the anatomical location of the liver, in CCl_4_-injured mice compared to the CSBD9 control conjugate (P < 0.01). Furthermore, the retention of [^89^Zr]Zr-DFO-C1–3 in CCl_4_ injured mice was 28% higher than the [^89^Zr]Zr-DFO-CSBD9 oil-treated group, although this did not reach statistical significance (Fig. 3B,C). This discrepancy can be explained due to the limitations associated with in vivo CLI quantification. The photons emitted during CLI are predominantly observed in the short-wavelength region of the visible light spectrum, below 500 nm, where light absorption by mouse biological tissue is high^40^. Consequently, factors such a tissue depth, signal attenuation by the surrounding tissues, and photon scatter all markedly reduce signal-to-noise ratio in CLI^41–44^.

To corroborate the in vivo imaging data, quantitative gamma counting analysis was subsequently performed on excised mouse tissues. The ex vivo biodistribution study indicated a significantly higher uptake of [^89^Zr]Zr-DFO-C1–3 in CCl_4_ injured liver (4.41 ± 0.19%ID/g) compared to all controls (Fig. 3D), while the primary signal was observed in the kidneys, consistent with the known renal clearance of scAbs (Supplementary Fig. S2B).

### C1–3 and CSBD9 CCl_4_ injured mice display the same level of injury

**Figure 4:**
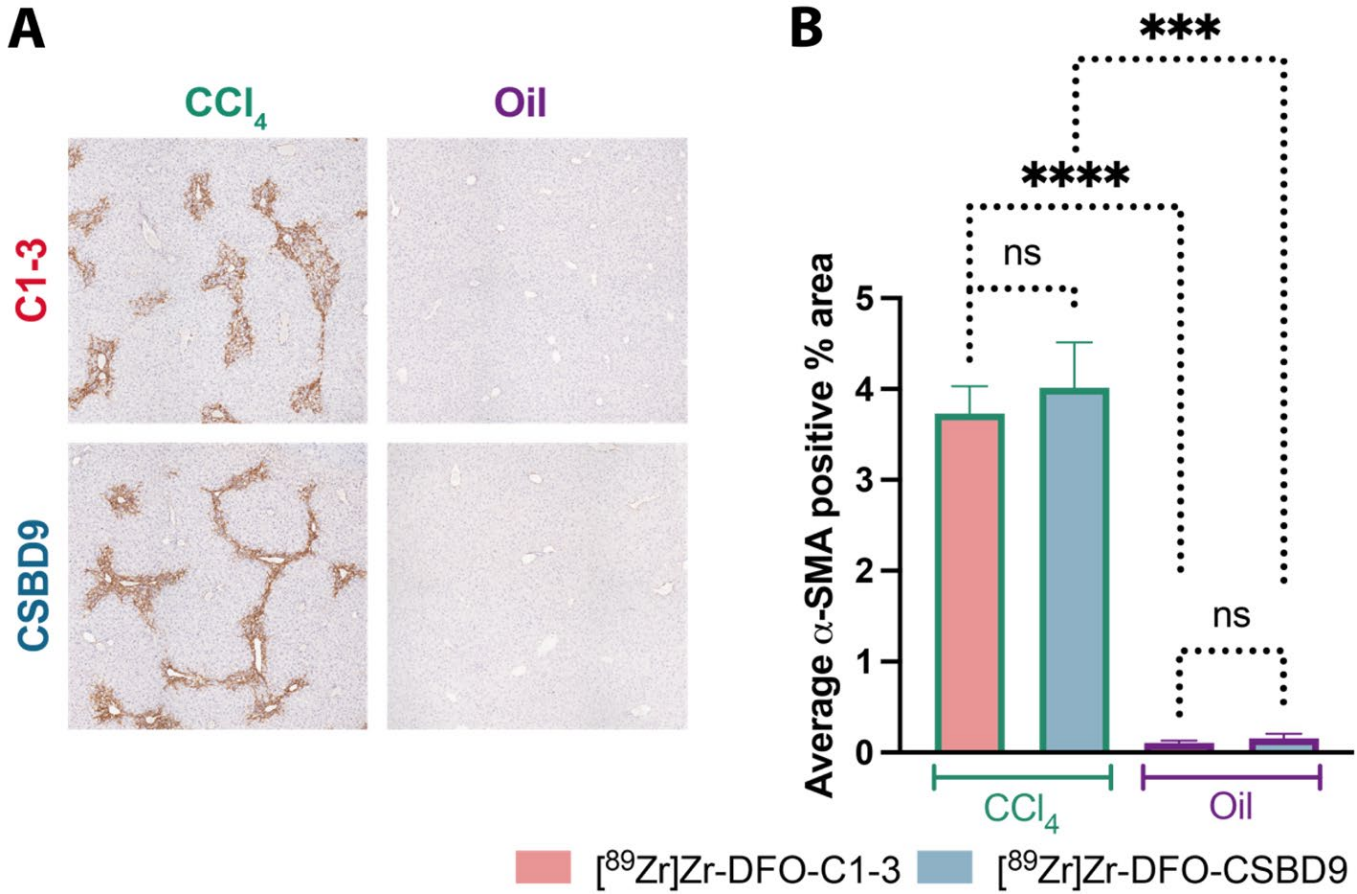
CCl_4_-injured mice display an equal number of HM. (**A**) Photomicrographs of α-SMA stained liver sections showing average α-SMA positive area (brown) in CCl_4_ (left) and oil (right) injured mice. (**B**) Quantified α-SMA positive percentage area. CCl_4_ causes significant activation of HM compared to oil. The number of HM is the same in the both [^89^Zr]Zr-DFO-C1–3 and [^89^Zr]Zr-DFO-CSBD9 administered mice. Data are mean ± SEM, minimum of n = 3.

To ascertain that the observed differences in in vivo and ex vivo signals were not due to differences in wound healing response and activation of HM, staining for alpha-smooth muscle actin (α-SMA), a marker of HM, was conducted on liver sections. The histological assessment showed that the area positive for α-SMA was comparable in both CCl_4_-injured groups. As expected, the oil-treated mice exhibited negligible expression of α-SMA, restricted to the vasculature (Fig. 4A,B).

## Discussion

In a healthy liver, HSCs exist in a quiescent state^45^. In response to hepatic injury, HSCs transdifferentiate into collagen-producing HMs to repair the tissue damage^46^, yet prolonged injury can lead HM to proliferate persistently, driving the progression from fibrosis to cirrhosis and potentially liver cancer^6,47^. The regenerative capabilities of the liver permit the reversal of fibrosis if the injury is diagnosed and treated at an early stage. However, the development of antifibrotics has been hindered by the lack of robust diagnostic tools to non-invasively and longitudinally monitor the progression of liver fibrosis and evaluate therapeutic responses^48^.

This study demonstrates the utility of a synaptophysin-specific radiolabelled single chain antibody ([^89^Zr]Zr-DFO-C1–3) for monitoring HM in vivo. Previously, we showed that a fluorescently-labelled C1–3 probe served as an effective in vivo diagnostic tool for monitoring HM using preclinical optical imaging. However, the clinical applicability of this methodology is limited due to significant attenuation of the fluorescence signal by endogenous tissues, resulting in quantification challenges in clinical environments. Even in mice, where the liver is approximately 1–1.5 cm in depth, photon detection can be impeded by tissue absorption. Photon scatter, skin pigmentation and autofluorescence are additional attributing factors that further reduce the fluorescence signal detected by the charged coupled device camera mounted on an optical imaging system^40^. This underscores the need for a tracer that is compatible with PET imaging as this technique offers limitless tissue depth penetration, high sensitivity, and is widely used in clinical settings. Due to the present lack of a preclinical PET scanner in our institute, we have instead leveraged the Cerenkov luminescence imaging (CLI) capabilities of an in vivo optical imaging system to monitor the biodistribution of [^89^Zr]Zr-DFO-C1–3. While CLI quantification is constrained by high tissue photon attenuation, it provides effective utility as a preclinical tool for rapid, cost-effective visualisation of PET tracer distribution^49^. This utility is further substantiated by the increasing utilisation of CLI in clinical imaging applications. A recent study by Pratt et al. demonstrated the ability of CLI to locate tumours in cancer patients with existing or suspected tumours with nodal metastases using five different radiotracers^50^. In addition, Chen et al*.* achieved a significant milestone by successfully implementing the first CLI system within a community-based hospital^51^.

The CLI approach has been instrumental in evaluating the potential of radiolabelled C1–3 as a candidate for PET imaging in tracking fibrogenesis. In our research, C1–3 was labelled with the positron-emitting isotope zirconium-89, facilitating non-invasive nuclear imaging to visualise HM. Consistent with prior findings, our results indicate that the clearance of C1–3 occurs within 24 h^30,34^; a factor that contributed to the selection of [^89^Zr]Zr due to its sufficiently long half-life of 3.3 days that provides an ample window for imaging the distribution and activity of C1–3 in relation to HM.

[^89^Zr]Zr-DFO-C1–3 and control scAb, [^89^Zr]Zr-DFO-CSBD9, were synthesised with high radiolabelling efficiencies and were found to have high solution radiostability. An HM cell internalisation assay indicated uptake of [^89^Zr]Zr-DFO-C1–3 was significantly higher compared to the control [^89^Zr]Zr-DFO-CSBD9, demonstrating that binding specificity for SYN was retained after the ^89^Zr-labelling process. Further enhancements in cellular internalisation of the C1–3 radiotracer may be achieved by modification with cell-penetrating peptides, or by modulating the charge distribution and lipophilicity of the scAb conjugate^52^.

In vivo Cerenkov luminescence data revealed that [^89^Zr]Zr-DFO-C1–3 was able to differentiate between CCl_4_ injured mice and oil-treated controls. The specificity of C1–3 was validated against the control scAb (CSBD9) to confirm that the retention of C1–3 was not due to the inability of the injured liver to clear [^89^Zr]Zr-DFO-C1–3 efficiently. Although the [^89^Zr]Zr-DFO-C1–3 signal was significantly higher compared to CCl_4_ injured mice injected with [^89^Zr]Zr-DFO-CSBD9 and olive oil-treated mice administered [^89^Zr]Zr-DFO-C1–3, no significant differences were observed in the uptake in oil-treated mice given [^89^Zr]Zr-DFO-CSBD9. Such discrepancies are not a surprise as in vivo Cerenkov signal detection is a semi-quantitative methodology that is significantly affected by tissue depth. However, we anticipate that PET will yield enhanced statistical power due to its high sensitivity and excellent depth penetration. Transition to PET imaging holds the promise of discerning such small biological differences. Nonetheless, to determine if this inconsistency was due to a lack of photons penetrating through tissue, we performed ex vivo gamma counting on the liver tissue. Gamma counting represents a robust, highly quantitative technique to assess radioactivity uptake^53,54^. Ex vivo gamma counting demonstrated that [^89^Zr]Zr-DFO-C1–3 significantly differentiated between liver disease and oil controls, as well as the control CSBD9. The predominant signal was detected in the kidneys, which is attributable to the renal excretion of single-chain antibodies (scAbs). To enhance the specificity of C1–3 detection, subsequent research will focus on the generation of nanobodies with increased binding affinity for the target antigen, SYN. Owing to their comparatively small molecular structure, nanobodies penetrate tissues more efficiently, ensuring potent binding to antigens that are otherwise inaccessible to larger constructs. We expect the reduced size of nanobodies will therefore lead to enhancedcontrast ratios^55^. Specifically for the C1–3 nanobody, those fragments that do not bind to the intended antigen are quickly processed through renal clearance, thereby producing a more distinct signal in the liver.

Our findings serve as a preliminary validation for the continued exploration of ^89^Zr-labelled C1–3 as a viable PET imaging agent for chronic liver diseases (CLD), ranging from fibrosis to cancer. Building on our laboratory’s previous work, which demonstrated the utility of fluorescently labelled C1–3 in tracking HM within a Bile Duct Ligation model of chronic liver injury^30^, the subsequent logical progression is to evaluate the sensitivity of [^89^Zr]Zr-DFO-C1–3 on the same model. Furthermore, C1–3 presents an opportunity to categorise liver cancers based on their fibrogenic composition, especially those rich in HM, thereby assisting in the tailored management of such conditions.

Recent advances in PET ring detectors and integration of PET with anatomical imaging modalities such as MRI and CT have significantly improved the quantification, resolution and visualisation of soft tissue such as the liver^56–58^. Therefore, ^89^Zr-labelled C1–3 PET imaging in conjunction with MRI elastography has the potential to provide dynamic characterisation of liver fibrosis, from HM activity to build-up of scar formation. PET imaging with [^89^Zr]Zr-DFO-C1–3 tracer has the promise to diagnose and monitor a therapeutic response in CLD.

In preclinical research PET imaging with [^89^Zr]Zr-DFO-C1–3 fulfils a commitment to the 3Rs as it eliminates the need use multiple animals to generate longitudinal data, and allows researchers to assess HM numbers as CLD progresses or determine the effect of antifibrotics that suppress HM proliferation or clearance.

C1–3 is a human single-chain antibody which effectively binds to human HM. Therefore, the imaging approach described here has the potential to 1 day benefit patient diagnosis and promptly assess the dynamic changes of hepatic fibrogenesis in response to therapeutics.

## Conclusions

The findings of this study highlight the promising utility of [^89^Zr]Zr-DFO-C1–3 as an effective in vivo imaging agent for non-invasive monitoring of hepatic myofibroblasts. Employing a mouse model to simulate acute liver injury, our research demonstrates that [^89^Zr]Zr-DFO-C1–3 is capable of distinguishing between injury and healthy liver tissues. Furthermore, this PET tracer represents a substantial improvement over our previously reported fluorescently-labelled variant as it offers unlimited tissue depth penetration, which is crucial for comprehensive liver imaging, and therefore helps to address a critical need in diagnosis and management of hepatic disease.

Nevertheless, a limitation of our study was the utilisation of CLI in lieu of preclinical PET which restricted more extensive exploration of the in vivo properties of the radiotracer. Despite this, [^89^Zr]Zr-DFO-C1–3 exhibits promise as a candidate for further optimisation and sets a useful foundation for future research aimed at developing effective probes for liver pathology diagnosis and monitoring.

### Supplementary Information

Supplementary Information.

## Data Availability

The datasets generated and material generated in the current study are available from the corresponding author on reasonable request.

## Abbreviations

α-SMA: Alpha smooth muscle actin
CCl_4_: Carbon tetrachloride
CL: Cerenkov luminescence
CLI: Cerenkov luminescence imaging
CPM: Counts per minute
CSBD9: Single chain antibody negative control
C1–3: Single chain antibody targeted to synaptophysin
DFO: Desferrioxamine
FLI: Fluorescence luminescent imaging
HCC: Hepatocellular carcinoma
HM: Hepatic myofibroblast
HSC: Hepatic stellate cells
iTLC: Instant thin layer chromatography
I.P.: Intraperitoneal
MALDI-TOF: Matrix-assisted laser desorption/ionization-time of flight
MRI: Magnetic resonance imaging
LFT: Liver function test
PET: Positron emission tomography
scAb: Single chain antibody
ScFv: Single chain variable fragment
SYN: Synaptophysin
^89^Zr: Zirconium-89
%ID/g: Percentage of injected dose per gram
