# Supplementary material for: Synthesis and preclinical evaluation of a 89Zr-labelled human single chain antibody for non-invasive detection of hepatic myofibroblasts in acute liver injury
Source: Sci Rep. 2024 Jan 5;14:633. doi: 10.1038/s41598-023-50779-w (PMC10770171; doi:10.1038/s41598-023-50779-w)
Supplement: Supplementary file 1 — Supplementary Information. [file 41598_2023_50779_MOESM1_ESM.pdf]

Supplementary Data

Supplementary Figure 1

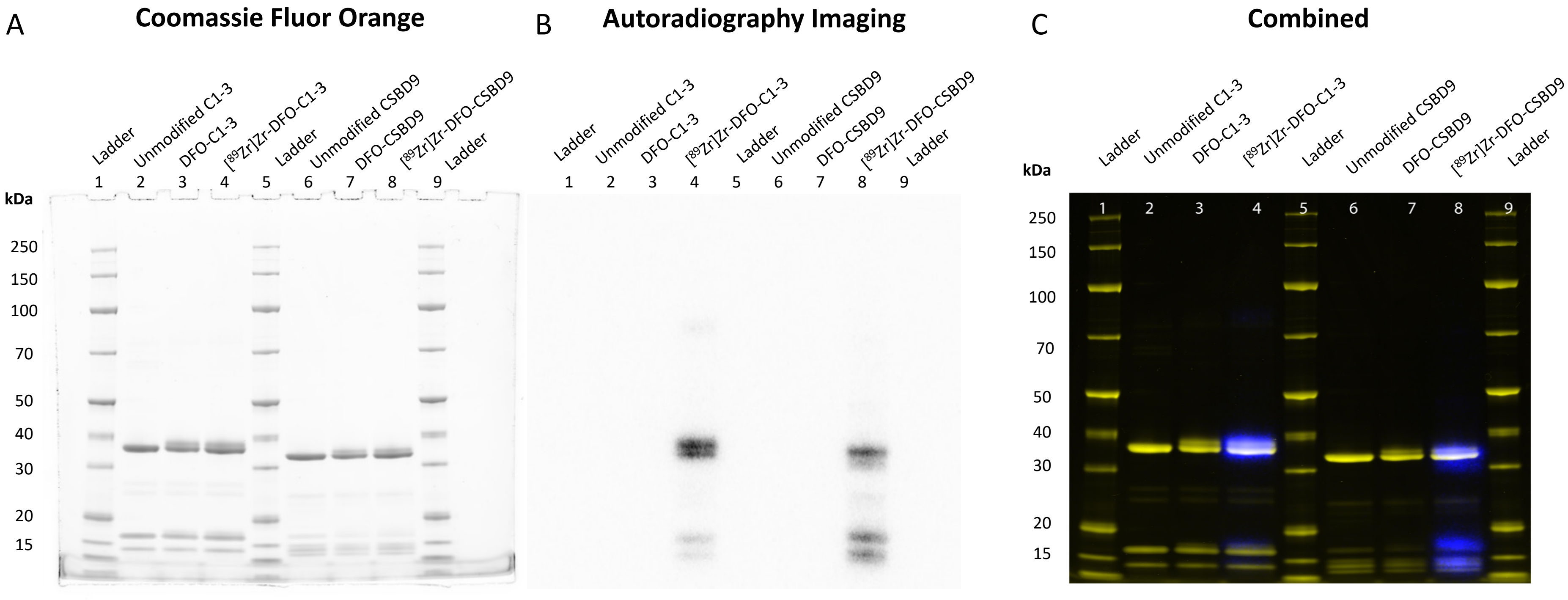

Supplementary figure 1. *Radio-SDS-PAGE of [^{89}Zr]Zr-DFO-scAb conjugates. (A) Total protein staining performed with Coomassie Flour Orange. (B) Autoradiography image, displaying radioactive signal from ^{89}Zr labelled single-chain antibodies only. (C) Combined data displaying colocalization of Coomassie Fluor Orange protein stain (yellow) and autoradiography (blue).*

Supplementary Figure 2

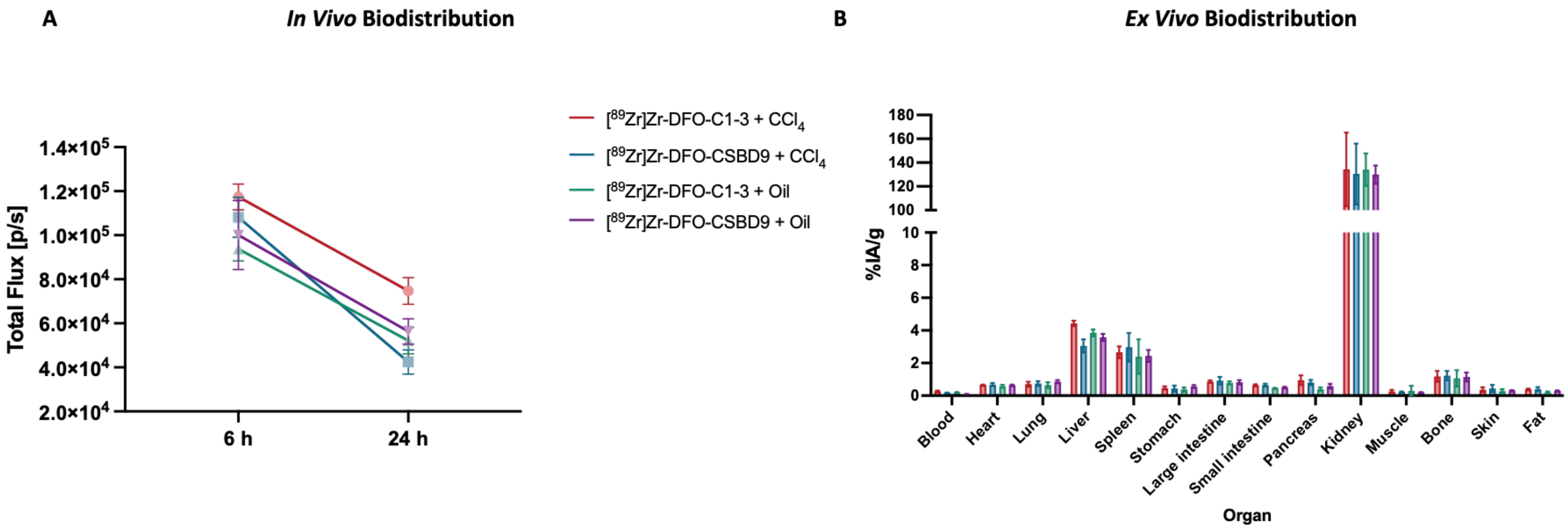

Supplementary figure 2. *A) In vivo biodistribution: Wild-type C57BL/6 mice were injured with an acute dose of* CCl_4 *(2 µL/g) to induce activation of HM and SYN expression. The control group was I.P. administered with olive oil, which does not provoke HM activation or expression of SYN. 48 h post-injury, mice were administered with 0.89 ± 0.04 MBq of either [*^{89}*Zr]Zr-DFO-C1-3 or [*^{89}*Zr]Zr-DFO-CSBD9 via IV injection followed by in vivo CLI at 6h and 24h***.** *A maximum signal is observed at 6h with a drop in signal detection as antibodies are cleared. B) Organ biodistribution: The* CCl_4-[89Zr]Zr-DFO-C1-3 is highly retained in the liver compared to all other groups/organs (except kidneys). *Due to the renal clearance of C1-3, the majority of the CL signal is observed in the kidneys.*

Supplementary Table 1

Radioactive doses and injected mass for each mouse.

Treatment	ScAb	Activity At Injection (MBq)	D.C. Residual (MBq)	Injected Dose (MBq)	Injected Mass (ug)
CCl_4	C1-3	0.92	0.08	0.84	9.32
CCl_4	C1-3	1.00	0.08	0.92	10.21
CCl_4	C1-3	1.02	0.11	0.91	10.10
CCl_4	C1-3	0.97	0.07	0.90	9.99
CCl_4	C1-3	0.95	0.08	0.87	9.66
CCl_4	CSBD9	1.00	0.07	0.93	10.32
CCl_4	CSBD9	0.94	0.09	0.85	9.43
CCl_4	CSBD9	0.96	0.09	0.87	9.66
CCl_4	CSBD9	1.04	0.07	0.97	10.77
CCl_4	CSBD9	0.99	0.07	0.92	10.21
Oil	C1-3	0.95	0.07	0.88	9.76
Oil	C1-3	0.98	0.07	0.91	10.10
Oil	C1-3	0.91	0.06	0.85	9.43
Oil	C1-3	0.94	0.05	0.89	9.87
Oil	CSBD9	0.90	0.11	0.79	8.77
Oil	CSBD9	0.93	0.07	0.86	9.54
Oil	CSBD9	0.97	0.07	0.90	9.99
Oil	CSBD9	0.94	0.06	0.88	9.77
